# Genetic and environmental influence on white matter: insight from an Italian twin population study

**DOI:** 10.3389/fnhum.2026.1765036

**Published:** 2026-03-18

**Authors:** Giovanni Videtta, Chiara Colli, Letizia Squarcina, Corrado Fagnani, Emanuela Medda, Cristina D’Ippolito, Carolina Bonivento, Maria Nobile, Paolo Brambilla

**Affiliations:** 1 Department of Neurosciences and Mental Health, Fondazione IRCCS Ca’ Granda, Ospedale Maggiore Policlinico, Milan, Italy; 2 Department of Pathophysiology and Transplantation, University of Milan, Milan, Italy; 3 Centre for Behavioural Sciences and Mental Health, Istituto Superiore di Sanità, Rome, Italy; 4 Scientific Institute IRCCS Eugenio Medea, Pasian di Prato, Udine, Italy; 5 Child and Adolescent Psychiatry Unit, Scientific Institute IRCCS Eugenio Medeai, Bosisio Parini, Lecco, Italy

**Keywords:** diffusion tensor imaging, genetics, heritability, tract-based spatial statistics, twins, white matter

## Abstract

**Introduction:**

The heritability of white matter (WM) has been a central focus of diffusion neuroimaging and genetic research. Twin studies have reported high heritability estimates for WM integrity, primarily based on fractional anisotropy (FA). However, other diffusion tensor imaging (DTI) metrics—mean diffusivity (MD), axial diffusivity (AD), and radial diffusivity (RD)—remain less explored.

**Methods:**

In the present twin study, we assessed WM heritability using FA, MD, AD, and RD metrics. Tract-Based Spatial Statistics was applied to DTI data from 81 healthy twin pairs (33 monozygotic, 48 dizygotic) recruited through the population-based Italian Twin Registry. Twin correlations and genetic and environmental variance components were estimated for each DTI index across brain regions.

**Results:**

Monozygotic twins exhibited higher correlations than dizygotic twins across most brain regions; however, a few regions showed an unexpected inverse pattern. Notably, while several regions demonstrated strong genetic influences, others showed no detectable genetic contribution, suggesting a substantial role for environmental factors in shaping WM characteristics in those areas.

**Conclusion:**

These findings revealed regional variability in WM heritability and challenged the assumption of uniform genetic influence, highlighting the importance of considering early environmental factors and supporting the development of more nuanced models of WM development.

## 1 Introduction

White matter (WM) is the main connective tissue which facilitates efficient communication between disparate regions of the human brain, underpinning a myriad of cognitive and behavioral functions (Bassett et al., 2011). Advancements in neuroimaging, particularly diffusion tensor imaging (DTI), have enabled researchers to non-invasively probe the microstructural integrity of WM tracts, offering insights into the neural substrates of various neuropsychiatric conditions and cognitive processes (Tournier et al., 2011). Among the DTI-derived metrics, fractional anisotropy (FA)—the degree of anisotropy within axonal fibers—has been extensively utilized to assess WM integrity, with studies consistently reporting moderate to high heritability estimates for FA across different age groups. For instance, research leveraging data from the Human Connectome Project and the ENIGMA-DTI consortium has demonstrated heritability estimates for FA ranging from 53 to 90%, underscoring a substantial genetic influence on WM microstructure (Kochunov et al., 2015).

In the last two decades, DTI has been employed to investigate the genetic and environmental influences on WM tissues in twin populations (Blokland et al., 2012). Twin studies indeed provide an excellent tool for analyzing the intricate relationship between genetic and environmental influences on traits of interest. By comparing monozygotic (MZ) and dizygotic (DZ) twins, researchers can estimate the heritability of various brain traits, including susceptibility to psychiatric disorders (Brambilla et al., 2014; Colli et al., 2024), the influence of genetic factors on behavior (Maggioni et al., 2024), and patterns of functional connectivity activation (Tassi et al., 2023). From this perspective, investigating WM heritability in twins provides valuable insights into the interplay between genetic and environmental influences on brain connectivity. This interaction underpins individual differences in cognition and behavior, as well as susceptibility to psychopathological conditions (Fagnani and Stazi, 2020; Maggioni et al., 2020). Twin studies have consistently demonstrated that DTI metrics are significantly influenced by genetic factors, underscoring their fundamental role in WM development. In neonatal populations, research has reported substantial heritability estimates for FA, radial diffusivity (RD) and axial diffusivity (AD)—which reflects diffusion perpendicular and parallel to axonal fibers, respectively—across several WM tracts, including fornix, cingulum and corpus callosum (Lee et al., 2015). These findings suggest that genetic factors play a critical role in the early development of WM microstructure, as supported by previous evidence (Gao et al., 2019), which has highlighted how the genetic architecture of WM is complex and highly polygenic (Arnatkeviciute et al., 2021; Zhao et al., 2021a). It is not surprising that large-scale genome-wide association studies (GWAS) have identified numerous genetic loci associated with variations in WM microstructure (Sha et al., 2023). For example, a study analyzing data from over 17,000 UK Biobank participants found that DTI parameters are substantially heritable across all WM tracts, with a mean heritability of 48.7%. Moreover, the study identified 213 independent significant single-nucleotide polymorphisms (SNPs) associated with 90 DTI parameters, many of which have been implicated in cognitive and mental health traits (Zhao et al., 2021b).

However, most studies investigating WM heritability in twin populations present several limitations that may affect the generalizability and interpretation of their findings (Videtta et al., 2024). First, research samples are often heterogeneous, frequently including unpaired twins or siblings, which can confound heritability estimates. Second, the reliance on specific datasets may reduce the representativeness of study populations. For example, commonly used datasets such as Vietnam Era Twin Study of Aging (VETSA) (Kremen et al., 2013) and Older Australian Twins Study (OATS) (Sachdev et al., 2009) are drawn from highly specific cohorts, including war veterans and older twin adults, respectively. Such sampling constraints may also introduce gender imbalances; notably, the VETSA dataset comprises only male twin pairs (Kremen et al., 2013). Additionally, while FA is widely used as a measure of WM integrity, other DTI metrics—such as mean diffusivity (MD), which reflects average water diffusion within axonal fibers, as well as AD and RD—remain underexplored in the context of heritability, despite offering complementary insights into WM microstructure.

In light of these limitations, the present study aims to estimate WM heritability across all major DTI indices (FA, MD, AD, RD) in a representative sample of healthy Italian twins. To strengthen current evidence, we combined Tract-Based Spatial Statistics (TBSS) with classical twin modeling, focusing on data from paired twin samples to ensure methodological consistency and demographic representativeness.

## 2 Method

### 2.1 Participants

A total sample of 121 pairs (242 twins) was selected from the twin cohorts participating in the “WhyMe?” and in the “SPES” Projects (Italian Ministry of Health, grants n. RF-2011-02352308, and n. GR-2010-2316745, respectively). Twins were recruited through the population-based Italian Twin Registry (Medda et al., 2019) in two different centers, Scientific Institute IRCCS Eugenio Medea of Bosisio Parini (Lecco, Italy) and Scientific Institute IRCCS Eugenio Medea of Pasian di Prato (Udine, Italy). Inclusion criteria were: (i) healthy twins; (ii) availability of Magnetic Resonance Imaging (MRI) scan; (iii) quality of DTI images acceptable for analyses. Unpaired twins and those pairs in which only one member met inclusion criteria were excluded. From 121 pairs (242 twins), 6 pairs (12 twins) were excluded due to psychiatric disorders in one or both twins and 19 pairs (38 twins) due to missing MRI data. An additional 15 pairs (30 twins) were ruled out due to low-quality DTI data, leading to a final sample of 81 pairs (162 twins): 47 pairs from the Scientific Institute IRCCS Eugenio Medea of Bosisio Parini (Lecco, Italy), and 34 pairs from the Scientific Institute IRCCS Eugenio Medea of Pasian di Prato (Udine, Italy).

The study was approved by the local ethical committee according to the Declaration of Helsinki and its subsequent versions (World Medical Association, 2013). Participants provided a written informed consent. For minors (<18 years), an assent was obtained, and a legal tutor provided informed consent.

### 2.2 MRI acquisition

Each participant underwent an MRI acquisition either at Scientific Institute IRCCS Eugenio Medea of Bosisio Parini (Lecco, Italy) or Scientific Institute IRCCS Eugenio Medea of Pasian di Prato (Udine, Italy). Two different MRI scanners were employed: Philips Achieva D-Stream 3T and Philips Achieva 3T. Acquisition protocols were identical across scanners. Diffusion images were acquired axially with anterior–posterior direction and one no-weighted image was acquired at *b* = 0 s/mm^2^: spin-echo sequencing, 64 directions, *b* = 1,000 s/mm^2^, echo time (TE) = 76 ms, repetition time (TR) = 8,895 ms, voxel size = 1.5 × 1.5 × 1.5, number of slices = 65, matrix = 138 × 138, field of view (FOV) = 207 × 207, flip angle = 90 degrees.

### 2.3 Tract-based spatial statistics

Diffusion-weighted images were preprocessed using tools from the FMRIB Software Library (FSL; Jenkinson et al., 2012). For each participant, diffusion data were corrected for eddy current-induced distortions and simple head motion using FSL’s *eddy_correct*, applying an affine registration of all diffusion-weighted volumes to the first b0 image. Non-brain tissue was removed using the Brain Extraction Tool (*BET*) with a fractional intensity threshold of 0.1, and the resulting brain mask was used for subsequent tensor fitting. Because the diffusion preprocessing was performed using *eddy_correct*, which does not include slice-wise motion correction or outlier replacement, residual motion effects, particularly relevant in pediatric and adolescent samples, could not be fully excluded. A diffusion tensor model was fitted at each voxel to compute the maps of diffusion scalar metrics: FA, MD, AD. Maps of RD were computed using FSL’s *fslmaths* by dividing the sum of the second (L2) and third (L3) eigenvalue maps by 2. Participants’ DTI maps were processed using Tract-Based Spatial Statistics (Smith et al., 2006). In detail, nonlinear registration was applied to align the images to the FMRIB58 FA template. Then, the registered images were averaged and thresholded at 0.2 to create a mean FA skeleton representing the central trajectories of WM tracts. Each participant’s DTI data were then projected onto this skeleton.

Regional diffusion metrics were extracted from the TBSS skeleton using a custom Python script based on Nibabel and NumPy (see Supplementary materials). Skeletonised diffusion maps (FA, MD, AD, RD) generated by TBSS were stored as 4D images in standard space, with the fourth dimension indexing participants. The Johns Hopkins University (JHU) ICBM-DTI-81 white matter atlas was used to define regions of interest (Mori et al., 2008). For each atlas-defined region, voxel indices corresponding to the region label were identified. For each participant, diffusion values were extracted from the skeletonised maps at those voxel locations. To ensure that only voxels belonging to the TBSS skeleton were included, zero-valued voxels (i.e., voxels outside the skeleton projection) were excluded from the computation. Mean diffusion values were then calculated across the remaining voxels within each region, yielding one regional value per participant per metric. Regions defined bilaterally in the atlas were analysed separately for left and right hemispheres. Six regions (middle cerebellar peduncle, pontine crossing tract, bilateral medial lemniscus, and bilateral inferior cerebellar peduncle) were excluded due to systematic quality issues and poor skeleton representation. Diffusion data quality was assessed following preprocessing and TBSS projection through visual inspection of corrected diffusion volumes, tensor-derived maps, and skeletonised images. Datasets showing severe motion artefacts, signal dropout, or excessive noise that compromised reliable tensor estimation or skeleton projection were excluded. Based on these criteria, 15 twin pairs were removed from the analysis. Exclusion rates did not differ between monozygotic and dizygotic twins, reducing the likelihood of zygosity-related bias.

Finally, ComBat harmonization (Fortin et al., 2017) was performed at the regional level on diffusion values extracted from the TBSS skeleton, rather than at the voxelwise level, to mitigate scanner-related site effects. Scanner model was specified as the batch variable, while age and sex were included as biological covariates to be preserved, ensuring that developmental and sex-related variability in WM microstructure was retained.

**Figure 1:**
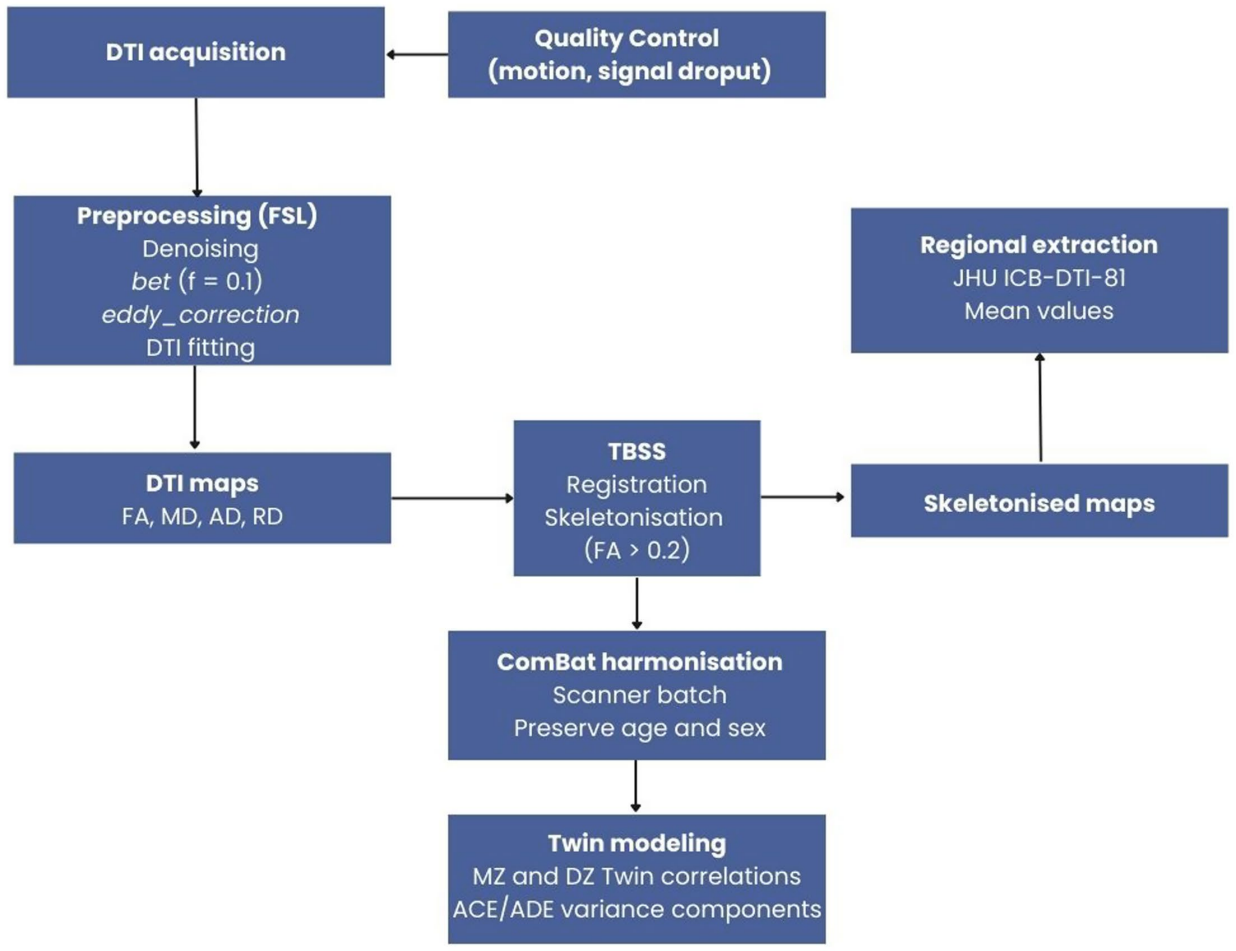
Overview of the diffusion MRI preprocessing and analysis pipeline. Diffusion-weighted images were preprocessed using FSL, followed by TBSS-based skeletonization. Regional diffusion metrics were extracted from the skeleton using the JHU ICBM-DTI-81 atlas, harmonized across sites using ComBat (preserving age and sex), and analyzed using twin modeling.

Figure 1 reports the schematic workflow of the diffusion MRI preprocessing and analysis pipeline.

### 2.4 Twin modeling

**Figure 2:**
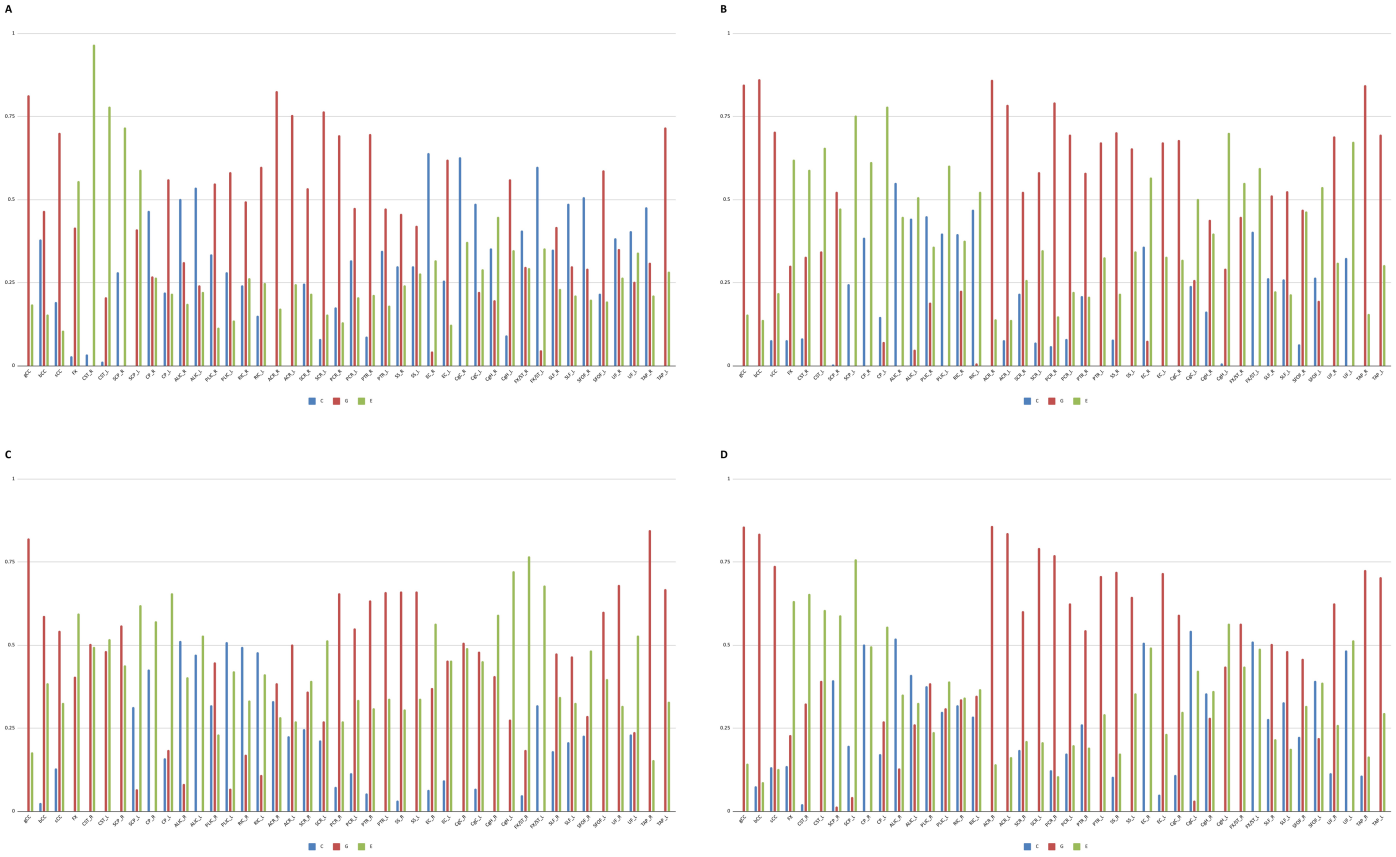
Genetic and environmental variance components for each diffusion tensor imaging metrics. **(A)** FA genetic and environmental variance components. **(B)** MD genetic and environmental variance components. **(C)** AD genetic and environmental variance components. **(D)** RD genetic and environmental variance components. C, Shared environment; G, Heritability [equal to additive (“narrow-sense”, A) heritability under the ACE model, or to additive plus non-additive (“broad-sense”, A + D) heritability under the ADE model]; E, unshared environment; gCC, Genu of Corpus Callosum; bCC, Body of Corpus Callosum; sCC, Splenium of Corpus Callosum; FX_R, Fornix Right; FX_L, Fornix Left; CST_R, Corticospinal Tract Right; CST_L, Corticospinal Tract Left; SCP_R, Superior Cerebral Peduncle Right; SCP_L, Superior Cerebral Peduncle Left; CP_R, Cerebral Peduncle Right; CP_L, Cerebral Peduncle Left; ALIC_R, Anterior Limb of Internal Capsule Right; ALIC_L, Anterior Limb of Internal Capsule Left; PLIC_R, Posterior Limb of Internal Capsule Right; PLIC_L, Posterior Limb of Internal Capsule Left; RIC_R, Retrolenticular part of Internal Capsule; RIC_L, Retrolenticular part of Internal Capsule Left; ACR_R, Anterior Corona Radiata Right; ACR_L, Anterior Corona Radiata Left; SCR_R, Superior Corona Radiata Right; SCR_L, Superior Corona Radiata Left; PCR_R, Posterior Corona Radiata Right; PCR_L, Posterior Corona Radiata Left; PTR_R, Posterior Thalamic Radiation Right; PTR_L, Posterior Thalamic Radiation Left; SS_R, Sagittal Stratum Right; SS_L, Sagittal Stratum Left; EC_R, External Capsule Right; EC_L, External Capsule Left; CgC_R, Cingulum (Cingulate gyrus) Right; CgC_L, Cingulum (Cingulate gyrus) Left; CgH_R, Cingulum (Hippocampus) Right; CgH_L, Cingulum (Hippocampus) Left; FX/ST_R, Fornix (cres)/Stria terminalis Right; FX/ST_L, Fornix (cres)/Stria terminalis Left; SLF_R, Superior Longitudinal Fasciculus Right; SLF_L, Superior Longitudinal Fasciculus Left; SFOF_R, Superior Fronto-Occipital Fasciculus Right; SFOF_L, Superior Fronto-Occipital Fasciculus Left; UF_R, Uncinate Fasciculus Right; UF_L, Uncinate Fasciculus Left; TAP_R, Tapetum Right; TAP_L, Tapetum Left.

Twin modeling analyses were performed with the Mx Software (Neale et al., 2006). Univariate biometric twin models incorporating additive genetic (A), either shared environmental (C) or non-additive genetic (D), and unshared (individual-specific) environmental (E) components of variance (Neale and Cardon, 1992) were applied to DTI metrics for the 42 WM regions by using sex- and age-adjusted correlations as input data for MZ and DZ groups. The A component represents the additive effects of all gene variants (i.e., alleles) that influence the trait, without interactive effects; the D component represents interactions between alleles at the same fixed chromosomal site (i.e., locus) (“dominance”) or at different loci (“epistasis”); the C component represents the effects of environmental factors that are shared by the twins within the family (e.g., rearing environment, family socio-economic status, parental behaviors) or in the womb (e.g., hormonal exposures); the E component represents the effects of environmental factors that are unique to an individual (e.g., lifestyles, relations with peers, infections), including measurement error. Only three of these four variance components can be simultaneously estimated because C and D components are confounded when analyzing data from MZ and DZ twins reared together. Given the limited power of the study, it was deemed prudent to report the estimated genetic and environmental components of variance for each of the regions and DTI indices under the fully parameterized (i.e., either ACE or ADE) models only, without looking for more parsimonious sub-models, which might penalize magnitude-relevant, yet not significant components. For the same reason, only point estimates of correlations in MZ and DZ pairs and of variance components were interpreted, because large standard errors often resulted in non-significant *p*-values, even in the case of sizeable correlation differences and variance components. The genetic component (G), referred to within the text and depicted in Figure 2, indicates the additive (“narrow-sense,” A) heritability under the ACE model, or the additive plus non-additive (“broad-sense,” A + D) heritability under the ADE model.

### 2.5 Statistical analysis

Statistical significance for gender and age differences was assessed using a Python script. A chi-squared (*χ*^2^) test was applied for gender, and an unpaired *t*-test for age. A significance threshold of *p* < 0.05 was used for both tests.

## 3 Results

### 3.1 Sociodemographic data

The sample consisted of 81 pairs (162 twins), including 78 males and 84 females, with a mean age of 16.7 ± 6.1 years (range: 9–32 years) (Supplementary Table S1). Of these, 33 were MZ pairs (18 males, 15 females; mean age: 16.8 ± 6.4 years) and 48 were DZ pairs (21 males, 27 females; mean age: 13.3 ± 2.5 years), of which 38 were same-sex pairs and 10 were opposite-sex pairs.

Comparing MZ and DZ pairs, no differences were found in gender (*χ*^2^ = 3.9, *p* = 0.27) and age (*t* = 0.14, *p* = 0.88) (Supplementary Table S1).

### 3.2 Twin correlations

Twin correlations were performed on MZ pairs and DZ pairs. Substantial correlations were observed across all DTI metrics, with MZ twins generally showing higher correlations than DZ twins in most brain regions (Table 1). Specifically, the mean correlations for MZ and DZ pairs, respectively, were: (i) FA: 0.71 (range: −0.07 to 0.91) and 0.49 (range: 0.07–0.65); (ii) MD: 0.58 (range: 0.15–0.89) and 0.37 (range: 0.01–0.57); (iii) AD: 0.56 (range: 0.08–0.85) and 0.39 (range: 0.06–0.57); and (iv) RD: 0.65 (range: 0.30–0.91) and 0.42 (range: 0.05–0.57).

**Table 1:** Twins correlations and genetic/environmental variance components for each DTI index.

Region	Twins correlations			Genetic and environmental variance components							
	MZ	DZ	*p*-value*	Model	A	C	D	A + D	G	E	Total
FA											
gCC	0.81	0.37	0.0006	ADE	0.65	–	0.17	0.81	0.81	0.19	1.00
bCC	0.85	0.61	0.0126	ACE	0.47	0.38	–	–	0.47	0.15	1.00
sCC	0.89	0.54	0.0002	ACE	0.70	0.19	–	–	0.70	0.11	1.00
FX	0.44	0.24	0.1578	ACE	0.42	0.03	–	–	0.42	0.56	1.00
CST_R	−0.02	0.07	0.3386	ACE	0.00	0.03	–	–	0.00	0.97	1.00
CST_L	0.22	0.12	0.3262	ACE	0.21	0.01	–	–	0.21	0.78	1.00
SCP_R	0.22	0.32	0.3201	ACE	0.00	0.28	–	–	0.00	0.72	1.00
SCP_L	0.41	0.16	0.1193	ADE	0.22	–	0.19	0.41	0.41	0.59	1.00
CP_R	0.73	0.60	0.1494	ACE	0.27	0.47	–	–	0.27	0.27	1.00
CP_L	0.78	0.50	0.0168	ACE	0.56	0.22	–	–	0.56	0.22	1.00
ALIC_R	0.81	0.66	0.0696	ACE	0.31	0.50	–	–	0.31	0.19	1.00
ALIC_L	0.78	0.66	0.1429	ACE	0.24	0.54	–	–	0.24	0.22	1.00
PLIC_R	0.88	0.61	0.0018	ACE	0.55	0.34	–	–	0.55	0.12	1.00
PLIC_L	0.86	0.57	0.0026	ACE	0.58	0.28	–	–	0.58	0.14	1.00
RIC_R	0.74	0.49	0.0417	ACE	0.49	0.24	–	–	0.49	0.26	1.00
RIC_L	0.75	0.45	0.0191	ACE	0.60	0.15	–	–	0.60	0.25	1.00
ACR_R	0.83	0.40	0.0006	ADE	0.75	–	0.08	0.83	0.83	0.17	1.00
ACR_L	0.75	0.30	0.0022	ADE	0.45	–	0.30	0.75	0.75	0.25	1.00
SCR_R	0.78	0.52	0.0202	ACE	0.54	0.25	–	–	0.54	0.22	1.00
SCR_L	0.85	0.46	0.0008	ACE	0.77	0.08	–	–	0.77	0.15	1.00
PCR_R	0.87	0.52	0.0007	ACE	0.69	0.18	–	–	0.69	0.13	1.00
PCR_L	0.79	0.56	0.0268	ACE	0.48	0.32	–	–	0.48	0.21	1.00
PTR_R	0.79	0.44	0.0059	ACE	0.70	0.09	–	–	0.70	0.21	1.00
PTR_L	0.82	0.58	0.0194	ACE	0.47	0.35	–	–	0.47	0.18	1.00
SS_R	0.76	0.53	0.0439	ACE	0.46	0.30	–	–	0.46	0.24	1.00
SS_L	0.72	0.51	0.0705	ACE	0.42	0.30	–	–	0.42	0.28	1.00
EC_R	0.68	0.66	0.4346	ACE	0.04	0.64	–	–	0.04	0.32	1.00
EC_L	0.88	0.57	0.0011	ACE	0.62	0.26	–	–	0.62	0.12	1.00
CgC_R	0.60	0.64	0.3861	ACE	0.00	0.63	–	–	0.00	0.37	1.00
CgC_L	0.71	0.60	0.2028	ACE	0.22	0.49	–	–	0.22	0.29	1.00
CgH_R	0.55	0.45	0.2866	ACE	0.20	0.35	–	–	0.20	0.45	1.00
CgH_L	0.65	0.37	0.0495	ACE	0.56	0.09	–	–	0.56	0.35	1.00
FX/ST_R	0.71	0.56	0.1438	ACE	0.30	0.41	–	–	0.30	0.29	1.00
FX/ST_L	0.65	0.62	0.4339	ACE	0.05	0.60	–	–	0.05	0.35	1.00
SLF_R	0.77	0.56	0.0513	ACE	0.42	0.35	–	–	0.42	0.23	1.00
SLF_L	0.79	0.64	0.0941	ACE	0.30	0.49	–	–	0.30	0.21	1.00
SFOF_R	0.80	0.65	0.0882	ACE	0.29	0.51	–	–	0.29	0.20	1.00
SFOF_L	0.81	0.51	0.0099	ACE	0.59	0.22	–	–	0.59	0.19	1.00
UF_R	0.73	0.56	0.0963	ACE	0.35	0.38	–	–	0.35	0.27	1.00
UF_L	0.66	0.53	0.2002	ACE	0.25	0.41	–	–	0.25	0.34	1.00
TAP_R	0.79	0.63	0.0861	ACE	0.31	0.48	–	–	0.31	0.21	1.00
TAP_L	0.72	0.32	0.0077	ADE	0.56	–	0.16	0.72	0.72	0.28	1.00
MD											
gCC	0.85	0.31	0.0000	ADE	0.39	–	0.46	0.85	0.85	0.15	1.00
bCC	0.86	0.38	0.0001	ADE	0.65	–	0.22	0.86	0.86	0.14	1.00
sCC	0.78	0.43	0.0062	ACE	0.70	0.08	–	–	0.70	0.22	1.00
FX	0.38	0.23	0.2404	ACE	0.30	0.08	–	–	0.30	0.62	1.00
CST_R	0.41	0.25	0.2171	ACE	0.33	0.08	–	–	0.33	0.59	1.00
CST_L	0.36	0.01	0.0602	ADE	0.00	–	0.34	0.34	0.34	0.66	1.00
SCP_R	0.53	0.26	0.0915	ADE	0.52	0.00	–	–	0.52	0.47	1.00
SCP_L	0.14	0.32	0.2209	ACE	0.00	0.25	–	–	0.00	0.75	1.00
CP_R	0.17	0.53	0.0389	ACE	0.00	0.39	–	–	0.00	0.61	1.00
CP_L	0.22	0.18	0.4369	ACE	0.07	0.15	–	–	0.07	0.78	1.00
ALIC_R	0.52	0.57	0.3827	ACE	0.00	0.55	–	–	0.00	0.45	1.00
ALIC_L	0.49	0.47	0.4463	ACE	0.05	0.44	–	–	0.05	0.51	1.00
PLIC_R	0.64	0.55	0.2660	ACE	0.19	0.45	–	–	0.19	0.36	1.00
PLIC_L	0.38	0.41	0.4426	ACE	0.00	0.40	–	–	0.00	0.60	1.00
RIC_R	0.62	0.51	0.2394	ACE	0.23	0.40	–	–	0.23	0.38	1.00
RIC_L	0.48	0.47	0.4921	ACE	0.01	0.47	–	–	0.01	0.52	1.00
ACR_R	0.86	0.42	0.0002	ADE	0.81	–	0.05	0.86	0.86	0.14	1.00
ACR_L	0.86	0.47	0.0004	ACE	0.78	0.08	–	–	0.78	0.14	1.00
SCR_R	0.74	0.48	0.0339	ACE	0.52	0.22	–	–	0.52	0.26	1.00
SCR_L	0.65	0.36	0.0445	ACE	0.58	0.07	–	–	0.58	0.35	1.00
PCR_R	0.85	0.46	0.0006	ACE	0.79	0.06	–	–	0.79	0.15	1.00
PCR_L	0.78	0.43	0.0071	ACE	0.70	0.08	–	–	0.70	0.22	1.00
PTR_R	0.79	0.50	0.0127	ACE	0.58	0.21	–	–	0.58	0.21	1.00
PTR_L	0.67	0.26	0.0097	ADE	0.36	–	0.31	0.67	0.67	0.33	1.00
SS_R	0.78	0.43	0.0061	ACE	0.70	0.08	–	–	0.70	0.22	1.00
SS_L	0.65	0.25	0.0123	ADE	0.34	–	0.32	0.65	0.65	0.35	1.00
EC_R	0.43	0.40	0.4232	ACE	0.08	0.36	–	–	0.08	0.57	1.00
EC_L	0.67	0.33	0.0219	ADE	0.64	–	0.04	0.67	0.67	0.33	1.00
CgC_R	0.68	0.16	0.0021	ADE	0.00	–	0.68	0.68	0.68	0.32	1.00
CgC_L	0.50	0.37	0.2499	ACE	0.26	0.24	–	–	0.26	0.50	1.00
CgH_R	0.60	0.38	0.1067	ACE	0.44	0.16	–	–	0.44	0.40	1.00
CgH_L	0.30	0.15	0.2560	ACE	0.29	0.01	–	–	0.29	0.70	1.00
FX/ST_R	0.45	0.15	0.0806	ADE	0.16	–	0.29	0.45	0.45	0.55	1.00
FX/ST_L	0.33	0.45	0.2630	ACE	0.00	0.40	–	–	0.00	0.60	1.00
SLF_R	0.78	0.52	0.0259	ACE	0.51	0.26	–	–	0.51	0.22	1.00
SLF_L	0.78	0.52	0.0212	ACE	0.53	0.26	–	–	0.53	0.22	1.00
SFOF_R	0.54	0.30	0.1112	ACE	0.47	0.07	–	–	0.47	0.46	1.00
SFOF_L	0.46	0.36	0.3084	ACE	0.20	0.27	–	–	0.20	0.54	1.00
UF_R	0.69	0.23	0.0049	ADE	0.24	–	0.45	0.69	0.69	0.31	1.00
UF_L	0.10	0.48	0.0379	ACE	0.00	0.33	–	–	0.00	0.67	1.00
TAP_R	0.84	0.42	0.0004	ADE	0.83	–	0.01	0.84	0.84	0.16	1.00
TAP_L	0.70	0.21	0.0031	ADE	0.15	–	0.54	0.70	0.70	0.30	1.00
AD											
gCC	0.82	0.35	0.0004	ADE	0.58	–	0.25	0.82	0.82	0.18	1.00
bCC	0.61	0.32	0.0518	ACE	0.59	0.03	–	–	0.59	0.39	1.00
sCC	0.67	0.40	0.0484	ACE	0.54	0.13	–	–	0.54	0.33	1.00
FX	0.41	0.17	0.1379	ADE	0.28	–	0.12	0.41	0.41	0.59	1.00
CST_R	0.50	0.15	0.0433	ADE	0.10	–	0.41	0.50	0.50	0.50	1.00
CST_L	0.50	0.02	0.0120	ADE	0.00	–	0.48	0.48	0.48	0.52	1.00
SCP_R	0.56	0.18	0.0268	ADE	0.14	–	0.42	0.56	0.56	0.44	1.00
SCP_L	0.38	0.35	0.4361	ACE	0.07	0.31	–	–	0.07	0.62	1.00
CP_R	0.34	0.49	0.2317	ACE	0.00	0.43	–	–	0.00	0.57	1.00
CP_L	0.34	0.25	0.3335	ACE	0.18	0.16	–	–	0.18	0.66	1.00
ALIC_R	0.60	0.55	0.3966	ACE	0.08	0.51	–	–	0.08	0.40	1.00
ALIC_L	0.41	0.51	0.2988	ACE	0.00	0.47	–	–	0.00	0.53	1.00
PLIC_R	0.77	0.54	0.0421	ACE	0.45	0.32	–	–	0.45	0.23	1.00
PLIC_L	0.58	0.54	0.4152	ACE	0.07	0.51	–	–	0.07	0.42	1.00
RIC_R	0.67	0.58	0.2764	ACE	0.17	0.50	–	–	0.17	0.33	1.00
RIC_L	0.59	0.53	0.3666	ACE	0.11	0.48	–	–	0.11	0.41	1.00
ACR_R	0.72	0.52	0.0887	ACE	0.38	0.33	–	–	0.38	0.28	1.00
ACR_L	0.73	0.48	0.0427	ACE	0.50	0.23	–	–	0.50	0.27	1.00
SCR_R	0.61	0.43	0.1461	ACE	0.36	0.25	–	–	0.36	0.39	1.00
SCR_L	0.48	0.35	0.2421	ACE	0.27	0.21	–	–	0.27	0.52	1.00
PCR_R	0.73	0.40	0.0165	ACE	0.66	0.07	–	–	0.66	0.27	1.00
PCR_L	0.67	0.39	0.0486	ACE	0.55	0.11	–	–	0.55	0.33	1.00
PTR_R	0.69	0.37	0.0265	ACE	0.63	0.05	–	–	0.63	0.31	1.00
PTR_L	0.66	0.18	0.0048	ADE	0.06	–	0.60	0.66	0.66	0.34	1.00
SS_R	0.69	0.36	0.0222	ACE	0.66	0.03	–	–	0.66	0.31	1.00
SS_L	0.66	0.24	0.0100	ADE	0.30	–	0.36	0.66	0.66	0.34	1.00
EC_R	0.44	0.25	0.1845	ACE	0.37	0.06	–	–	0.37	0.56	1.00
EC_L	0.55	0.32	0.1154	ACE	0.45	0.09	–	–	0.45	0.45	1.00
CgC_R	0.51	0.20	0.0648	ADE	0.29	–	0.22	0.51	0.51	0.49	1.00
CgC_L	0.55	0.31	0.1038	ACE	0.48	0.07	–	–	0.48	0.45	1.00
CgH_R	0.41	0.13	0.0976	ADE	0.10	–	0.31	0.41	0.41	0.59	1.00
CgH_L	0.28	0.13	0.2582	ADE	0.24	–	0.03	0.28	0.28	0.72	1.00
FX/ST_R	0.23	0.14	0.3422	ACE	0.18	0.05	–	–	0.18	0.77	1.00
FX/ST_L	0.17	0.42	0.1261	ACE	0.00	0.32	–	–	0.00	0.68	1.00
SLF_R	0.66	0.42	0.0746	ACE	0.48	0.18	–	–	0.48	0.34	1.00
SLF_L	0.67	0.44	0.0719	ACE	0.47	0.21	–	–	0.47	0.33	1.00
SFOF_R	0.52	0.37	0.2235	ACE	0.29	0.23	–	–	0.29	0.48	1.00
SFOF_L	0.60	0.28	0.0405	ADE	0.50	–	0.10	0.60	0.60	0.40	1.00
UF_R	0.68	0.26	0.0077	ADE	0.34	–	0.34	0.68	0.68	0.32	1.00
UF_L	0.47	0.35	0.2705	ACE	0.24	0.23	–	–	0.24	0.53	1.00
TAP_R	0.85	0.33	0.0001	ADE	0.46	–	0.39	0.85	0.85	0.15	1.00
TAP_L	0.67	0.24	0.0080	ADE	0.28	–	0.39	0.67	0.67	0.33	1.00
RD											
gCC	0.86	0.31	0.0000	ADE	0.37	–	0.49	0.86	0.86	0.14	1.00
bCC	0.91	0.49	0.0000	ACE	0.84	0.08	–	–	0.84	0.09	1.00
sCC	0.87	0.50	0.0004	ACE	0.74	0.13	–	–	0.74	0.13	1.00
FX	0.37	0.25	0.2953	ACE	0.23	0.14	–	–	0.23	0.63	1.00
CST_R	0.35	0.18	0.2286	ACE	0.32	0.02	–	–	0.32	0.65	1.00
CST_L	0.41	0.04	0.0465	ADE	0.00	–	0.39	0.39	0.39	0.61	1.00
SCP_R	0.41	0.40	0.4854	ACE	0.01	0.40	–	–	0.01	0.59	1.00
SCP_L	0.24	0.22	0.4607	ACE	0.04	0.20	–	–	0.04	0.76	1.00
CP_R	0.38	0.59	0.1245	ACE	0.00	0.50	–	–	0.00	0.50	1.00
CP_L	0.44	0.31	0.2507	ACE	0.27	0.17	–	–	0.27	0.56	1.00
ALIC_R	0.65	0.58	0.3283	ACE	0.13	0.52	–	–	0.13	0.35	1.00
ALIC_L	0.67	0.54	0.1866	ACE	0.26	0.41	–	–	0.26	0.33	1.00
PLIC_R	0.76	0.57	0.0665	ACE	0.39	0.38	–	–	0.39	0.24	1.00
PLIC_L	0.61	0.45	0.1781	ACE	0.31	0.30	–	–	0.31	0.39	1.00
RIC_R	0.66	0.49	0.1410	ACE	0.34	0.32	–	–	0.34	0.34	1.00
RIC_L	0.63	0.46	0.1448	ACE	0.35	0.28	–	–	0.35	0.37	1.00
ACR_R	0.86	0.37	0.0001	ADE	0.63	–	0.23	0.86	0.86	0.14	1.00
ACR_L	0.84	0.42	0.0006	ADE	0.83	–	0.01	0.84	0.84	0.16	1.00
SCR_R	0.79	0.49	0.0117	ACE	0.60	0.19	–	–	0.60	0.21	1.00
SCR_L	0.79	0.40	0.0026	ADE	0.79	–	0.00	0.79	0.79	0.21	1.00
PCR_R	0.89	0.51	0.0001	ACE	0.77	0.12	–	–	0.77	0.11	1.00
PCR_L	0.80	0.49	0.0081	ACE	0.63	0.17	–	–	0.63	0.20	1.00
PTR_R	0.81	0.53	0.0133	ACE	0.55	0.26	–	–	0.55	0.19	1.00
PTR_L	0.71	0.34	0.0119	ADE	0.64	–	0.07	0.71	0.71	0.29	1.00
SS_R	0.83	0.46	0.0023	ACE	0.72	0.10	–	–	0.72	0.18	1.00
SS_L	0.65	0.28	0.0207	ADE	0.47	–	0.18	0.65	0.65	0.35	1.00
EC_R	0.50	0.51	0.4714	ACE	0.00	0.51	–	–	0.00	0.49	1.00
EC_L	0.77	0.41	0.0070	ACE	0.72	0.05	–	–	0.72	0.23	1.00
CgC_R	0.70	0.40	0.0314	ACE	0.59	0.11	–	–	0.59	0.30	1.00
CgC_L	0.58	0.56	0.4588	ACE	0.03	0.54	–	–	0.03	0.42	1.00
CgH_R	0.64	0.50	0.1876	ACE	0.28	0.36	–	–	0.28	0.36	1.00
CgH_L	0.44	0.18	0.1152	ADE	0.29	–	0.14	0.44	0.44	0.57	1.00
FX/ST_R	0.56	0.25	0.0529	ADE	0.45	–	0.12	0.56	0.56	0.44	1.00
FX/ST_L	0.51	0.51	0.4968	ACE	0.00	0.51	–	–	0.00	0.49	1.00
SLF_R	0.78	0.53	0.0253	ACE	0.50	0.28	–	–	0.50	0.22	1.00
SLF_L	0.81	0.57	0.0203	ACE	0.48	0.33	–	–	0.48	0.19	1.00
SFOF_R	0.68	0.45	0.0719	ACE	0.46	0.22	–	–	0.46	0.32	1.00
SFOF_L	0.61	0.50	0.2478	ACE	0.22	0.39	–	–	0.22	0.39	1.00
UF_R	0.74	0.43	0.0179	ACE	0.63	0.11	–	–	0.63	0.26	1.00
UF_L	0.34	0.59	0.0873	ACE	0.00	0.48	–	–	0.00	0.52	1.00
TAP_R	0.84	0.47	0.0017	ACE	0.73	0.11	–	–	0.73	0.17	1.00
TAP_L	0.70	0.20	0.0022	ADE	0.10	–	0.60	0.70	0.70	0.30	1.00

MZ, Monozygotic; DZ, Dizygotic; A, Additive genetics; C, Shared environment; D, Non-additive genetics; G, Heritability [equal to additive (“narrow-sense”, A) heritability under the ACE model, or to additive plus non-additive (“broad-sense”, A + D) heritability under the ADE model]; E, unshared environment; gCC, Genu of Corpus Callosum; bCC, Body of Corpus Callosum; sCC, Splenium of Corpus Callosum; FX_R, Fornix Right; FX_L, Fornix Left; CST_R, Corticospinal Tract Right; CST_L, Corticospinal Tract Left; SCP_R, Superior Cerebral Peduncle Right; SCP_L, Superior Cerebral Peduncle Left; CP_R, Cerebral Peduncle Right; CP_L, Cerebral Peduncle Left; ALIC_R, Anterior Limb of Internal Capsule Right; ALIC_L, Anterior Limb of Internal Capsule Left; PLIC_R, Posterior Limb of Internal Capsule Right; PLIC_L, Posterior Limb of Internal Capsule Left; RIC_R, Retrolenticular part of Internal Capsule; RIC_L, Retrolenticular part of Internal Capsule Left; ACR_R, Anterior Corona Radiata Right; ACR_L, Anterior Corona Radiata Left; SCR_R, Superior Corona Radiata Right; SCR_L, Superior Corona Radiata Left; PCR_R, Posterior Corona Radiata Right; PCR_L, Posterior Corona Radiata Left; PTR_R, Posterior Thalamic Radiation Right; PTR_L, Posterior Thalamic Radiation Left; SS_R, Sagittal Stratum Right; SS_L, Sagittal Stratum Left; EC_R, External Capsule Right; EC_L, External Capsule Left; CgC_R, Cingulum (Cingulate gyrus) Right; CgC_L, Cingulum (Cingulate gyrus) Left; CgH_R, Cingulum (Hippocampus) Right; CgH_L, Cingulum (Hippocampus) Left; FX/ST_R, Fornix (cres)/Stria terminalis Right; FX/ST_L, Fornix (cres)/Stria terminalis Left; SLF_R, Superior Longitudinal Fasciculus Right; SLF_L, Superior Longitudinal Fasciculus Left; SFOF_R, Superior Fronto-Occipital Fasciculus Right; SFOF_L, Superior Fronto-Occipital Fasciculus Left; UF_R, Uncinate Fasciculus Right; UF_L, Uncinate Fasciculus Left; TAP_R, Tapetum Right; TAP_L, Tapetum Left. ^*^One-tailed Fisher’s test of correlations.

**Figure 3:**
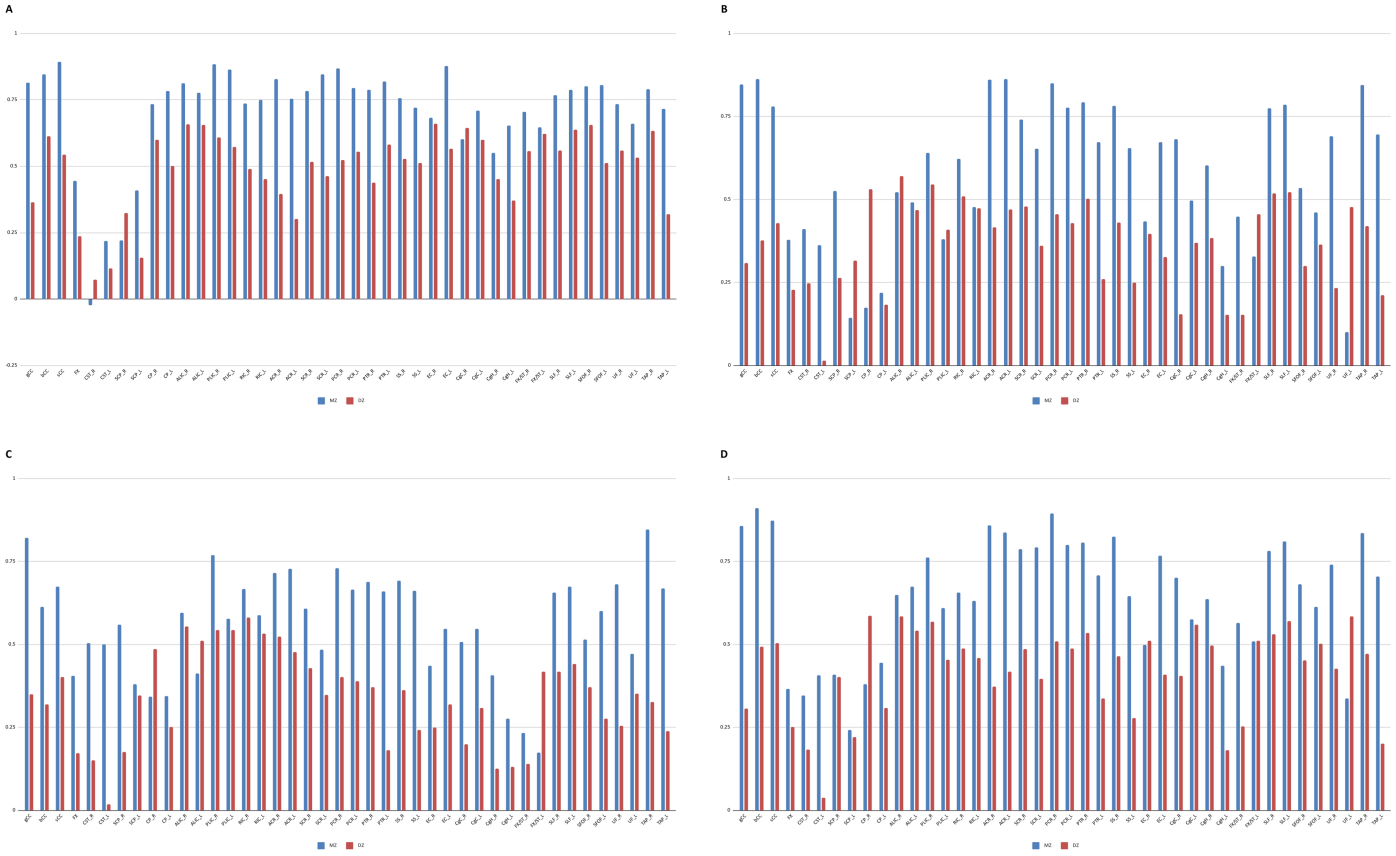
Twin correlations for each diffusion tensor imaging metrics. **(A)** FA twin correlations. **(B)** MD twin correlations. **(C)** Twin correlations for each diffusion tensor imaging metrics. AD twin correlations. **(D)** RD twin correlations. MZ, Monozygotic; DZ, Dizygotic; gCC, Genu of Corpus Callosum; bCC, Body of Corpus Callosum; sCC, Splenium of Corpus Callosum; FX_R, Fornix Right; FX_L, Fornix Left; CST_R, Corticospinal Tract Right; CST_L, Corticospinal Tract Left; SCP_R, Superior Cerebral Peduncle Right; SCP_L, Superior Cerebral Peduncle Left; CP_R, Cerebral Peduncle Right; CP_L, Cerebral Peduncle Left; ALIC_R, Anterior Limb of Internal Capsule Right; ALIC_L, Anterior Limb of Internal Capsule Left; PLIC_R, Posterior Limb of Internal Capsule Right; PLIC_L, Posterior Limb of Internal Capsule Left; RIC_R, Retrolenticular part of Internal Capsule; RIC_L, Retrolenticular part of Internal Capsule Left; ACR_R, Anterior Corona Radiata Right; ACR_L, Anterior Corona Radiata Left; SCR_R, Superior Corona Radiata Right; SCR_L, Superior Corona Radiata Left; PCR_R, Posterior Corona Radiata Right; PCR_L, Posterior Corona Radiata Left; PTR_R, Posterior Thalamic Radiation Right; PTR_L, Posterior Thalamic Radiation Left; SS_R, Sagittal Stratum Right; SS_L, Sagittal Stratum Left; EC_R, External Capsule Right; EC_L, External Capsule Left; CgC_R, Cingulum (Cingulate gyrus) Right; CgC_L, Cingulum (Cingulate gyrus) Left; CgH_R, Cingulum (Hippocampus) Right; CgH_L, Cingulum (Hippocampus) Left; FX/ST_R, Fornix (cres)/Stria terminalis Right; FX/ST_L, Fornix (cres)/Stria terminalis Left; SLF_R, Superior Longitudinal Fasciculus Right; SLF_L, Superior Longitudinal Fasciculus Left; SFOF_R, Superior Fronto-Occipital Fasciculus Right; SFOF_L, Superior Fronto-Occipital Fasciculus Left; UF_R, Uncinate Fasciculus Right; UF_L, Uncinate Fasciculus Left; TAP_R, Tapetum Right; TAP_L, Tapetum Left.

An unexpected finding from the analysis was that, in some brain regions, DZ twins showed higher correlations than MZ twins—albeit in some cases only marginally (Figure 3). For FA, this pattern was observed in the right corticospinal tract (CST_R) (MZ: −0.02, DZ: 0.07), right superior cerebellar peduncle (SCP_R) (MZ: 0.22, DZ: 0.32), and right cingulum (cingulate gyrus) (CgC_R) (MZ: 0.60, DZ: 0.64) (Figure 3A). A similar trend emerged for MD in the left superior cerebellar peduncle (SCP_L) (MZ: 0.14, DZ: 0.32), right cerebral peduncle (MZ: 0.17, DZ: 0.53), right anterior limb of the internal capsule (ALIC_R) (MZ: 0.52, DZ: 0.57), left posterior limb of the internal capsule (PLIC_L) (MZ: 0.38, DZ: 0.41), left fornix(cres)/stria terminalis (FX/ST_L) (MZ: 0.33, DZ: 0.46), and left uncinate fasciculus (UF_L) (MZ: 0.10, DZ: 0.48) (Figure 3B). For AD, DZ correlations exceeded those of MZ twins in the right cerebral peduncle (CP_R) (MZ: 0.34, DZ: 0.49), left anterior limb of internal capsule (ALIC_L) (MZ: 0.41, DZ: 0.51), and FX/ST_L (MZ: 0.17, DZ: 0.42) (Figure 3C). Finally, for RD, higher correlations in DZ twins were observed in the CP_R (MZ: 0.38, DZ: 0.59), right external capsule (EC_R) (MZ: 0.50, DZ: 0.51), FX/ST_L (MZ: 0.50, DZ: 0.51), and UF_L (MZ: 0.34, DZ: 0.59) (Figure 3D).

### 3.3 Genetic and environmental variance components

Genetic and environmental variance components were estimated on the same pairs of twins.

For each DTI metric, some regions were more sensitive to genetic variation (G), while others were more influenced by the environment, either shared (C) or not shared (E) (Table 1).

Interestingly, a few brain regions exhibited G component values close to zero, indicating a negligible genetic contribution (Figure 2). This pattern was observed for FA in the CST_R (C: 0.03, G: 0.00, E: 0.97), SCP_R (C: 0.28, G: 0.00, E: 0.72), and CgC_R (C: 0.63, G: 0.00, E: 0.37) (Figure 2A). For MD, regions with a null G component included the SCP_L (C: 0.25, G: 0.00, E: 0.75), CP_R (C: 0.39, G: 0.00, E: 0.61), ALIC_R (C: 0.55, G: 0.00, E: 0.45), PLIC_L (C: 0.40, G: 0.00, E: 0.60), FX/ST_L (C: 0.40, G: 0.00, E: 0.60), and UF_L (C: 0.33, G: 0.00, E: 0.67) (Figure 2B). A similar trend was seen for AD in the CP_R (C: 0.43, G: 0.00, E: 0.57), ALIC_L (C: 0.47, G: 0.00, E: 0.53), and FX/ST_L (C: 0.32, G: 0.00, E: 0.68) (Figure 2C). For RD, the same pattern was found in the CP_R (C: 0.50, G: 0.00, E: 0.50), EC_R (C: 0.51, G: 0.00, E: 0.49), FX/ST_L (C: 0.51, G: 0.00, E: 0.49), and UF_L (C: 0.48, G: 0.00, E: 0.52) (Figure 2D).

Conversely, several regions demonstrated a strong genetic (G) contribution across all or most DTI metrics (Figure 2). Notably, high G values were consistently observed in the genu (gCC) (FA: 0.82, MD: 0.85, AD: 0.82, RD: 0.86) and splenium (sCC) (FA: 0.70, MD: 0.70, AD: 0.54, RD: 0.74) of the corpus callosum, right anterior corona radiata (ACR_R) (FA: 0.83, MD: 0.86, AD: 0.38, RD: 0.86), left anterior corona radiata (ACR_L) (FA: 0.76, MD: 0.80, AD: 0.50, RD: 0.84), right posterior corona radiata (PTR_R) (FA: 0.70, MD: 0.79, AD: 0.66, RD: 0.77), right tapetum (TAP_R) (FA: 0.31, MD: 0.84, AD: 0.85, RD: 0.73), and left tapetum (TAP_L) (FA: 0.72; MD: 0.70; AD: 0.70; RD: 0.71).

## 4 Discussion

The present study found higher twin correlations in MZ compared to DZ twins across most WM regions; however, a few regions showed an inverse pattern. Notably, while some WM regions exhibited strong genetic contributions across all or most DTI metrics, several others demonstrated minimal genetic influence, as indicated by genetic (G) component values approaching zero.

### 4.1 Evidence of genetic and environmental divergence

Results of twin correlations are in line with the existing literature, which describes higher correlations for MZ than DZ twins for both FA (Bohlken et al., 2014) and MD (Elman et al., 2017). Less evidence has been reported for AD and RD, which seem to follow a correlational pattern similar to that of FA and MD, at least in corpus callosum and its subregions—gCC, body (bCC) and sCC—(Kanchibhotla et al., 2014). Our findings align with a well-established body of literature indicating that WM integrity is significantly influenced by genetic factors (Jahanshad et al., 2013), likely reflecting inherited variability in brain development (Schmitt et al., 2008). However, the observation of similar or even lower twin correlations in MZ compared to DZ twins in certain WM regions was unexpected.

This atypical pattern of similarity might reflect the influence of environmental factors overshadowing genetic contributions in specific brain regions. If DZ twins experience more similar environments than MZ twins—due to contextual or social factors—shared environmental exposures could elevate DZ correlations (Beam and Turkheimer, 2013). This less common scenario may arise when MZ twins differentiate themselves more in response to social or identity pressures, while DZ twins maintain more similar behaviors (Tucker-Drob and Bates, 2016). Environmental contributions to WM development have been further emphasized by evidence that socioeconomic status (SES) moderates heritability estimates. For instance, higher SES has been linked to increased genetic contributions to FA, suggesting that enriched environments might allow genetic potential to manifest more fully (Chiang et al., 2011). Taken together, these findings suggest that atypical patterns in twin correlations may arise from a complex interplay of genetic and environmental factors. However, these patterns should be interpreted with extreme caution and should be validated in larger and more diverse twin samples, in order to determine whether they can reflect broader biological principles or they are mainly attributable to sample-specific measurement noise or insufficient power. Furthermore, it should be recognized that the small sample size may have provided low power to disentangle A and C components for some of the regions analyzed; this may have led to inflated shared environmental estimates for these regions, which may be difficult to reconcile biologically.

### 4.2 Role of environment in white matter development

One of the most intriguing findings of the present study is the absence of genetic influence in certain WM regions across multiple DTI metrics. This observation contrasts with most existing literature, which consistently reports at least moderate heritability for WM traits in twin populations. A possible explanation is that genetic effects on WM microstructure may vary across developmental stages. For example, in a study of twins aged 12–29 years, Chiang et al. (2011) found that FA increased during adolescence, with heritability estimates being higher in adolescents than in adults. However, the authors did not compare narrower age groups within adolescence, leaving unresolved the possibility that genetic contributions may gradually increase over shorter developmental windows—such as between early and late adolescence—rather than emerging uniformly across the entire stage. This hypothesis is supported by additional evidence suggesting that genetic influences on WM development may not be fully expressed during late childhood or early adolescence, becoming more dominant in later phases of maturation. Brouwer et al. (2012) reported a longitudinal increase in heritability estimates for FA and a corresponding decrease in environmental influence between the ages of 9 and 12, specifically in the bilateral fornix and uncinate fasciculus (UF). However, these findings are region-specific and have not been widely replicated, limiting their generalizability. Despite this, they raise the possibility that environmental factors may temporarily compensate for or mask genetic influences during key developmental windows (Maggioni et al., 2020). In our study, the absence of genetic effects was not uniform across the brain; it was restricted to specific DTI metrics and localized to either the left or right hemisphere. Similar lateralized patterns have been observed in opposite-sex DZ twins (Luo et al., 2022). Luo et al. found that genetic contributions to MD and AD varied between hemispheres, particularly in the UF and cerebral peduncle. This hemispheric asymmetry may reflect specialized developmental trajectories that differentially engage genetic mechanisms. A compelling example of this phenomenon is the arcuate fasciculus, a tract involved in language processing. In the left hemisphere, where linguistic functions are dominant, the AF shows stronger genetic contributions, while in the right hemisphere, genetic effects appear to diminish, possibly due to reduced functional relevance and increased environmental modulation (Budisavljevic et al., 2015). Similarly, in the ventral cingulum, heritability estimates for FA and MD were reported at 32 and 22%, respectively, on the left side, compared to 0 and 1% on the right (Budisavljevic et al., 2016).

Although our results showed differences in heritability between hemispheres that may be related to WM asymmetries and lateralization processes, these findings should be interpreted with caution due to limited statistical power. Further larger and longitudinal twin studies are needed to better clarify the dynamic and region-specific influences of genes and environment on WM.

### 4.3 Limitations

To correctly interpret our results, several limitations should be considered. First, the relatively small number of twin pairs conferred low statistical power in twin modeling and related inferential issues. In particular, it was not possible to estimate genetic and environmental variance components on WM microstructure with adequate precision, and thus information about the relative importance of these components for the targeted brain measures could only be derived from point estimates. Clearly, the small sample size limited our ability not only to detect genetic influences for some brain regions where the models provided null heritability estimates, but also to stratify the sample and examine possible changes of WM heritability estimates in specific subgroups; in particular, it was not possible to test if these estimates vary across sexes by fitting sex-limitation models, or if they vary across specific developmental periods. Related to power, it should also be acknowledged that saturated model fitting for twin assumption checks was not feasible; however, preliminary sample descriptives showed only minor differences in the magnitude of means and variances across twin order and zygosity, thus providing some support to the validity of ACE modeling.

Second, potential confounding variables that may affect WM—such as nicotine use, non-addictive drinking behaviors (e.g., binge drinking), and psychiatric comorbidities among twins’ siblings—were not controlled for in the analysis. Third, our sample was predominantly composed of adolescent twins, who are still undergoing WM maturation, which may have influenced the findings. Finally, limitations in MRI data quality led to a reduced sample size and the exclusion of certain regions of interest, precluding heritability estimation in those areas.

## 5 Conclusion

Our study offered novel insights into the genetic and environmental contributions to WM microstructure in a representative sample of Italian twins. While many WM regions displayed substantial heritability, others showed minimal or no genetic influence, highlighting a significant role for environmental factors, including prenatal and developmental contexts. The regional and hemispheric variability in heritability patterns underscores the complex interplay between genetic and environmental influences on WM development. Despite limitations related to sample size, the findings enrich our understanding of WM architecture in non-clinical populations and emphasize the need for longitudinal, age- and sex-balanced twin studies to further clarify these dynamic processes.

## Data Availability

The original contributions presented in the study are included in the article/Supplementary material, further inquiries can be directed to the corresponding author.

## Glossary

gCC: Genu of Corpus Callosum
bCC: Body of Corpus Callosum
sCC: Splenium of Corpus Callosum
FX: Fornix
CST_R: Corticospinal Tract Right
CST_L: Corticospinal Tract Left
SCP_R: Superior Cerebral Peduncle Right
SCP_L: Superior Cerebral Peduncle Left
CP_R: Cerebral Peduncle Right
CP_L: Cerebral Peduncle Left
ALIC_R: Anterior Limb of Internal Capsule Right
ALIC_L: Anterior Limb of Internal Capsule Left
PLIC_R: Posterior Limb of Internal Capsule Right
PLIC_L: Posterior Limb of Internal Capsule Left
RIC_R: Retrolenticular part of Internal Capsule Right
RIC_L: Retrolenticular part of Internal Capsule Left
ACR_R: Anterior Corona Radiata Right
ACR_L: Anterior Corona Radiata Left
SCR_R: Superior Corona Radiata Right
SCR_L: Superior Corona Radiata Left
PCR_R: Posterior Corona Radiata Right
PCR_L: Posterior Corona Radiata Left
PTR_R: Posterior Thalamic Radiation Right
PTR_L: Posterior Thalamic Radiation Left
SS_R: Sagittal Stratum Right
SS_L: Sagittal Stratum Left
EC_R: External Capsule Right
EC_L: External Capsule Left
CgC_R: Cingulum (Cingulate gyrus) Right
CgC_L: Cingulum (Cingulate gyrus) Left
CgH_R: Cingulum (Hippocampus) Right
CgH_L: Cingulum (Hippocampus) Left
FX/ST_R: Fornix (cres)/Stria terminalis Right
FX/ST_L: Fornix (cres)/Stria terminalis Left
SLF_R: Superior Longitudinal Fasciculus Right
SLF_L: Superior Longitudinal Fasciculus Left
SFOF_R: Superior Fronto-Occipital Fasciculus Right
SFOF_L: Superior Fronto-Occipital Fasciculus Left
UF_R: Uncinate Fasciculus Right
UF_L: Uncinate Fasciculus Left
TAP_R: Tapetum Right
TAP_L: Tapetum Left
