# Supplementary material for: Genetic and environmental influence on white matter: insight from an Italian twin population study
Source: Front Hum Neurosci. 2026 Mar 18;20:1765036. doi: 10.3389/fnhum.2026.1765036 (PMC13045875; doi:10.3389/fnhum.2026.1765036)
Supplement: Supplementary file 1 [file Data_Sheet_1.docx]

**Genetic and environmental influence on white matter:**

**Insight from an Italian twin population study**

Giovanni Videtta, Chiara Colli, Letizia Squarcina, Corrado Fagnani et al.

**Supplementary materials**

**Python script**

import numpy as np

import nibabel as nib

import pandas as pd

import os

# Load the atlases and results

atl_path = 'JHU-ICBM-labels-1mm.nii.gz'

atl = nib.load(atl_path)

labels = atl.get_fdata()

results_path = 'your/path/TBSS_results' # Adjust path as needed

resultsh = nib.load(results_path)

results = resultsh.get_fdata()

# Define subject labels manually

roi_labels = ["ROI_1", “ROI_2”, “ROI_3”] # Adjust list with region labels of your atlas

# Ensure the list matches the number of ROIs

if len(roi_labels) != num_rois:

raise ValueError(f'ROI labels list has {len(roi_labels)} entries, expected {num_rois}')

# Define subject labels manually

subject_labels = ["Subject_1", "Subject_2", "Subject_3"] # Modify the name of subjects according to the dataset. Remember, YOU MUST FOLLOW

# THE ORDER OF SUBJECTS WITHIN TBSS FILE!!!!!!!

# Optional: Save NIfTI files for each ROI's covariate matrix

# nifti_output = nib.Nifti1Image(matrice_results_cov, affine=resultsh.affine)

# nib.save(nifti_output, f'all_FA_label_{i}.nii.gz')

# Initialize the tables for mean and standard deviation

tabella_medie = np.zeros((num_rois, num_subjects))

tabella_std = np.zeros((num_rois, num_subjects))

# Iterate over each ROI

for i in range(1, num_rois + 1): # MATLAB indices start at 1

indici = np.where(labels == i) # Find voxel indices for the ROI

for j in range(num_subjects):

valpat = results[..., j]

vettore = valpat[indici]

# Compute mean and std for non-zero elements

non_zero_vettore = vettore[vettore != 0]

if non_zero_vettore.size > 0:

tabella_medie[i - 1, j] = np.mean(non_zero_vettore)

tabella_std[i - 1, j] = np.std(non_zero_vettore)

# Convert to pandas DataFrames with proper row/column labels

medie_df = pd.DataFrame(tabella_medie, index=roi_labels, columns=subject_labels)

std_df = pd.DataFrame(tabella_std, index=roi_labels, columns=subject_labels)

# Save the data to an Excel file

output_file = 'roi_statistics.xlsx'

with pd.ExcelWriter(output_file) as writer:

medie_df.to_excel(writer, sheet_name='Mean Values')

std_df.to_excel(writer, sheet_name='Standard Deviations')

print(f'Excel file "{output_file}" saved successfully!')

**Supplementary Tables**

**Supplementary Table S1.** Sociodemographic characteristics of all twin pairs.

|  | **Twin sample (N = 81)** | | |
| --- | --- | --- | --- |
|  | **MZ (N = 33)** | **DZ (N = 48)** | **p-value** |
| **Sex (M:F**) | 18:15 | 21:27 | 0.27 |
| **Age**  **(mean ± SD)** | 16.8 ± 6.4 | 13.3 ± 2.5 | 0.88 |
| **Zygosity (SS:OS)** | - | 38:10 | - |

MZ: Monozygotic; DZ: Dizygotic; M: Males; F: Females; SD: Standard Deviation; SS: Same-Sex; OS: Opposite-Sex.

**Supplementary Figures**

**Supplementary Figure S1.** Representative diffusion MRI preprocessing and TBSS-based regional analysis workflow (axial view).


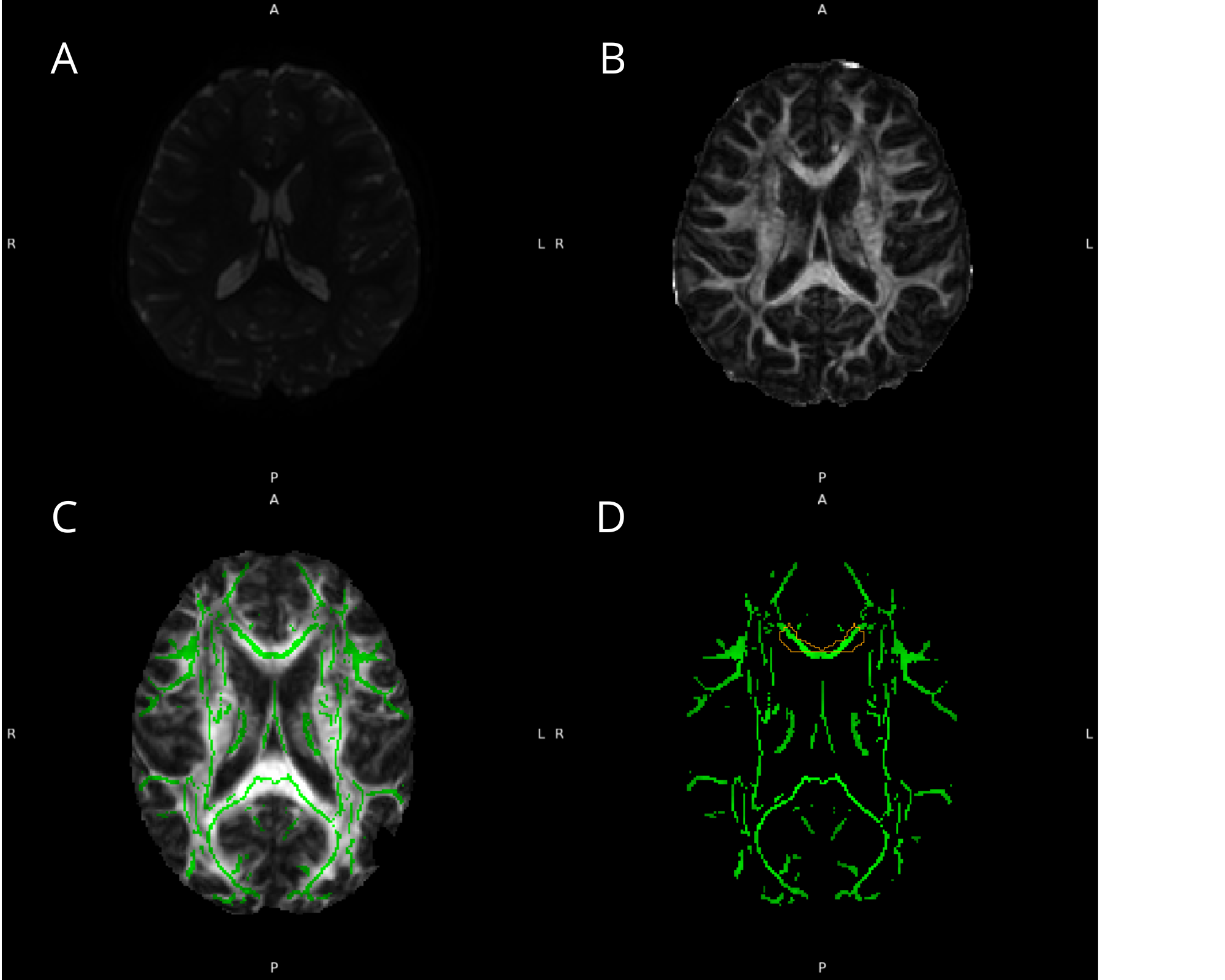


**(A)** Raw diffusion-weighted b0 image from a representative participant. **(B)** Fractional anisotropy (FA) map after preprocessing, including eddy-current and motion correction and brain extraction. **(C)** Mean FA skeleton generated using Tract-Based Spatial Statistics (TBSS), thresholded at FA > 0.2 and overlaid on the standard FA template. **(D)** Example of region-wise extraction in skeleton space, showing the intersection between the TBSS FA skeleton and a single white matter region from the JHU ICBM-DTI-81 atlas (genu of the corpus callosum). Regional diffusion metrics were computed by averaging non-zero skeleton voxels within each atlas-defined region. A: Anterior; P: Posterior; L: Left; R:Right.
